# A methodology for creating and validating psychological stories for conveying and measuring psychological traits

**DOI:** 10.1007/s11257-019-09219-6

**Published:** 2019-03-19

**Authors:** Kirsten A. Smith, Matt Dennis, Judith Masthoff, Nava Tintarev

**Affiliations:** 1 University of Southampton, Southampton, UK; 2 University of Portsmouth, Portsmouth, UK; 3 University of Aberdeen, Aberdeen, UK; 4 Utrecht University, Utrecht, Netherlands; 5 TU Delft, Delft, Netherlands

**Keywords:** Empirical methodology, Personality, Personality measurement, Research tools

## Abstract

Personality impacts all areas of our lives; it governs who we are and how we react to life’s challenges. Personalized systems that adapt to end users should take into account the user’s personality to perform well. Several methodologies (e.g. User-as-Wizard, indirect studies) that use personality adaptation require first for personality to be conveyed to the participant; this has few validated approaches. Furthermore, measuring personality is often time consuming, prone to response bias (e.g. using questionnaires) or data intensive (e.g. using behaviour or text mining). This paper presents a methodology for creating and validating stories to convey psychological traits and for using such stories with a *personality slider* scale to measure these traits. We present the validation of the scale and evaluate its reliability. To evidence the validity of the methodology, we outline studies where the stories and scale have been effectively applied (in recommender systems, intelligent tutoring systems, and persuasive systems).

## Introduction

Personality—“a person’s nature or disposition; the qualities that give one’s character individuality”^1^—is a key area of research in user modelling and user adaptive systems. One of the most popular ways to describe and measure personality is *trait theory*—where a person is assessed against one or more factors (e.g. ‘Conscientiousness’ or ‘Agreeableness’). These measurable differences in how people interact with the world are prime targets for providing users with an appropriately tailored user experience. However, to facilitate these tailored user experiences, researchers first need to discover which aspects of personality are important for adaptation, and how to tailor experience to them.^2^

One approach would be to measure users’ personality and ask them to use the system or evaluate its features. However, as noted in Paramythis et al.’s (2010) discussion on layered evaluation, one issue with using a user-based study for an adaptive system is that adaptation takes time, often more than is available during a study. One solution they advocate is an *indirect* study, where the user model is given to participants and they perform the task on behalf of a third party. This allows researchers to control the characteristics of the imaginary user, avoiding the time delay needed for populating the user model from actual user interactions with the system. An indirect study also ensures that the input to an adaptation layer is perfect, making it very suitable for layered evaluations. Indirect studies may also be required for other reasons—for example, they are needed when it is difficult to recruit a large enough number of target participants, such as in the work by Smith et al. (2016) for skin cancer patients.

Another way to investigate adaptation strategies and discover pertinent personality traits is by using a *User-as-wizard* approach (Masthoff 2006; Paramythis et al. 2010), which uses human behaviour to inspire the algorithms needed in an adaptive system. In a User-as-Wizard study, participants are given the same information the system would have, and are asked to perform the system’s task. Normally, participants will deal with fictional users, which allows us to study multiple participants dealing with the same user, controlling exactly what information participants get.

When using a User-as-Wizard or indirect approach for adaptation to personality research, the simulated user’s personality needs to be conveyed. However, there is a paucity of easy, validated ways to convey or represent the personality of a third party to participants. One option is to use real people, allowing participants to interact with a person with the desired trait. However, this is hard to control as it is hard to ensure participants adapt to personality instead of, for example, current affective state. Participants would have to spend considerable time with the individual to perceive their personality. Another option is to ask participants to “imagine a user who is extravert” or provide statements such as “John is neurotic”. This approach is unlikely to elicit empathy from participants due to a lack of context about the simulated user and could possibly be overlooked when placed with other data, such as test scores.

This is a non-trivial research problem: how to provide enough information about the personality of a simulated user for participants to identify and empathise with them, without making the simulated user seem one-dimensional and implausible. This paper details a methodology for conveying personality using validated *personality stories*.

In addition to conveying personality, these stories can be used as part of an alternative method of measuring personality.

Reliable and efficient personality measurement is still largely an open challenge. Whilst validated personality tests exist, completing them may create an overhead that is unacceptable to users: personality tests range from the Five Item Personality Inventory (FIPI test) (Gosling et al. 2003) to the 300-item International Personality Item Pool (IPIP-NEO) (Goldberg et al. 2006). A problem with questionnaires is response bias, in particular, the bias introduced by acquiescence or ‘yea-saying’—the tendency of individuals to consistently agree with survey items regardless of their content (Jackson and Messick 1958). This is an issue with many personality trait questionnaires, and was one reason why a new version of the Big Five Inventory (BFI-2) was produced recently (Soto and John 2017). Questionnaires may also be undesirable for reasons described later. Current approaches to unobtrusively measure personality include analysis of blogs (e.g. Nowson and Oberlander 2007; Iacobelli et al. 2011), users’ social media content (e.g. Facebook, Twitter) (Gao et al. 2013; Golbeck et al. 2011; Quercia et al. 2011) or social media behaviour (e.g. Amichai-Hamburger and Vinitzky 2010; Ross et al. 2009). These indirect approaches are however still far less reliable than direct approaches.

Using the personality stories as a basis, we propose an alternative and light-weight approach for reliably measuring personality, using so-called personality sliders with the stories at the slider ends, which is faster than completing many personality tests. We describe how identification with the people in personality stories can easily and engagingly be used to measure user personality. Personality sliders provide a broad characterisation of a personality trait, whilst at the same time making it less salient to participants what they are asked about. Personality sliders take about a minute to complete per trait (assuming an average reading speed), so are fast to administer and may save time particularly:

- In studies or systems that require a user characteristic for which short questionnaires do not yet exist. Short questionnaires only exist for some personality traits (most noticeably the Five Factor Model), whilst the slider approach can be used for any personality trait as well as other user characteristics. Of course, the personality stories are created from questionnaire items, and using more items increases reading time. However, only one decision/interaction is required per trait (compared to one per item for the questionnaires), reducing cognitive load and decision time.
- In studies that require both the measurement of the participants’ personality and the portrayal of the personality of fictional people—e.g. looking at the impact of self-similar personality on book recommendations for fictional users. Participants only need to read the stories once, so 1 min suffices to both complete the personality test and portray two fictional users’ personality.
- In studies or systems that require obtaining personality measurements for multiple people provided by one person. For example, in Moncur et al. (2014), automated messages about babies in intensive care to their parents’ social network were adapted to individual receivers’ characteristics. This may require a parent to indicate the emotional stability of the people closest to them. Using the personality sliders, participants only have to read the stories once, and then only need to make one decision/interaction per personality trait per person.

Another advantage of using personality sliders is that it reduces response bias. Using the personality story sliders, participants need to judge which person they resemble more, so are not agreeing/disagreeing with individual items, removing bias due to acquiescence. Multi-item surveys also tend to suffer from *straight-lining*. Straight-lining occurs when participants give identical (or nearly identical) responses to items in a battery of questions using the same response scale (Zhang and Conrad 2014). Requiring only one interaction per trait (as in the sliders) mitigates this. Finally, personality sliders provide a higher granularity of personality, as the sliders provide continuous rather than interval data, whilst most personality tests are restricted to a small number of points. This also means that the data is more appropriate for parametric analysis than traditional likert data.

To evidence the practical value of our methodology for conveying and measuring personality, we show how the personality stories and personality sliders have been successfully used in many of our studies (see Sect. 6).

**Fig. 1:**
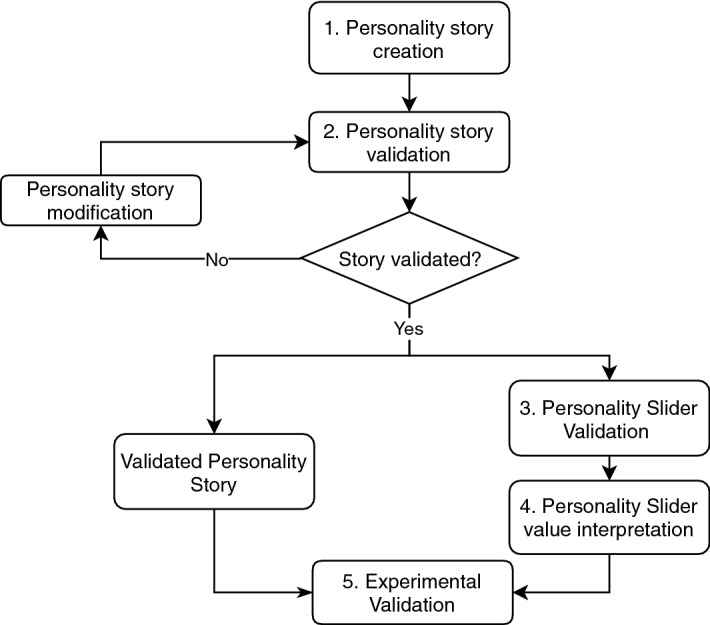
The methodology used in this paper for personality slider development

### Overview of methodology

Our methodology for conveying and measuring personality traits using personality stories (see Fig. 1) consists of the following stages:

1. Creating short stories about a person to express distinct personality traits (their *target trait*): we use Resilience, Generalized Self-Efficacy, and those from the Five Factor model.
2. Iteratively validating the generated stories to ensure that the stories convey their target trait at high and low levels, and are able to robustly portray the desired trait by asking people to fill out a personality questionnaire for the person in the story (different from the questionnaires used for story creation). Issues include both the case where the perceived score for a non-target trait (a personality trait other than the target trait) differs significantly between high and low story, and where the scores for these non-target traits lie outside a normative range. The pilots were conducted in the lab with later studies conducted using crowdsourcing for broader generalizability.
3. Validating the approach of measuring personality through stories by allowing users to pick which individual they are most like, using a slider. The values of these results were correlated with standardized personality tests for the same traits.
4. Outline how the slider values can be used to distinguish groups of users with distinct levels of personality traits. Before the sliders could be used in a system, or even applied experimentally to evaluate adaptation, we needed to define how to use the slider values. We summarise the advantages and disadvantages of the respective methods.
5. Validating the approach in an experiment where personality is likely to affect adaptation (i.e. use the stories in an experiment where you hypothesize that there ought to be an effect of personality). We tested the approach in multiple studies.

### Crowd sourcing participants

We rely heavily on rapid questionnaire responses from a participant pool to iteratively validate personality stories. Where the number of unique participants required was small, we used convenience sampling. However, our participant pool was too small for Five Factor Model validation as many iterations were required (explained in Sect. 4.3). To expand our participant pool, we decided to use the crowd-sourcing service, *Amazon Mechanical Turk* (MT) (2012).

MT is helpful when requiring large numbers of participants for studies. However, valid concerns exist that data collected online may be of lower quality and requires robust validation methods. Many studies, such as those described by Weinberg et al. (2014) have tried to show the validity of using MT to collect research data. These studies have generally found that the quality of MT data is comparable to what would be collected from supervised lab experiments, if studies are carefully set up, explained, and controlled. We follow recommended best practice in our MT experimental design and procedures.

In our work we have obtained some insights into using crowd-sourcing to gather experimental data. We were initially concerned that crowd-sourced participants (*workers*) would simply complete questionnaires in a random fashion in order to be paid. However, we found no evidence for this. “Gaming the system” by random scoring did not occur: participants correctly identified the personality trait we were portraying.

MT holds statistics on each *worker*, including *acceptance rate*. This is available to all *requesters* (those setting tasks) representing the percentage of work submitted by a particular worker that was approved (by all requesters). Thus if somebody consistently submits poor work, their acceptance rate drops. As requesters can set a high acceptance rate as a qualification for their tasks, this causes participants to value their acceptance rate, and complete tasks conscientiously. In addition to this, the integrated Cloze Test for English Fluency (Taylor 1953) was used as an attentional check to ensure participants were carefully reading the instructions, and had enough literacy skills to understand the task. We were also able to restrict participation to the United States only, which considerably drops the possibility of spam in the results.

The paper is structured as follows. Section 2 surveys the literature on measuring, conveying and adapting to personality. Section 3 describes the story creation process. Section 4 discusses the process of story validation. In Sect. 5, we test using the stories to measure user personality and outline how these results can be applied to group users by personality trait. Section 6 shows the application of the methodology by summarising many studies that investigated adaptation to personality and used the stories to convey or measure personality. Section 7 concludes the paper, discusses its limitations and provides directions for future work.

## Related work

In this section, we describe the models of personality used in this paper and the rationale for choosing these, focusing specifically on trait theories and social learning approaches. We summarize the methods for obtaining users’ personality traits and then summarize how personality can be portrayed, building on these methods. Finally, we discuss adaptation to personality in recommender systems, persuasive systems, and intelligent tutoring systems. We focus on adaptation to particular personality traits and the acquisition and portrayal of personality in the studies conducted.

**Table 1:** The five robust dimensions of personality from Fiske (1949) to present Reproduced from Digman (1990)

Author	I	II	III	IV	V
Fiske (1949)	Social adaptivity	Conformity	Will to achieve	Emotional control	Inquiring intellect
Eysenck (2013)	Extraversion	<————Psychoticism————>		Neuroticism	
Tupes and Christal (1992)	Surgency	Agreeableness	Dependability	Emotionality	Culture
Norman (1963)	Surgency	Agreeableness	Conscientiousness	Emotional	Culture
Borgatta (1964)	Assertiveness	Likeability	Task interest	Emotionality	Intelligence
Cattell (1957)	Exvia	Cortertia	Superego strength	Anxiety	Intelligence
Guilford (1975)	Social activity	Paranoid disposition	Thinking introversion	Emotional stability	
Digman (1988)	Extraversion	Friendly compliance	Will to achieve	Neuroticism	Intellect
Hogan (1986)	Sociability and ambition	Likeability	Prudence	Adjustment	Intellectance
Costa and McCrae (1985)	Extraversion	Agreeableness	Conscientiousness	Neuroticism	Openness
Peabody and Goldberg (1989)	Power	Love	Work	Affect	Intellect
Buss and Plomin (1984)	Activity	Sociability	Impulsivity	Emotionality	
Tellegen (1985)	Positive emotionality		Constraint	Negative emotionality	
Lorr (1986)	Interpersonal involvement	Level of socialization	Self-control	Emotional stability	Independent

### Models of personality

#### Personality trait theories

Traits are defined as “an enduring personal characteristic that reveals itself in a particular pattern of behaviour in different situations” (Carlson et al. 2004, p. 583). Over time, trait theorists have tried to identify and categorise these traits (Carlson et al. 2004). The number of traits identified has varied, with competing theories arising. The best known include Eysenck’s three factors (Eysenck 2013), Cattell’s 16PF (Cattell 1957), and the Five-Factor Model (FFM) (Goldberg 1993). More recently a general consensus towards five main traits (or dimensions) (Digman 1990; McCrae and John 1992) has emerged, shown in Table 1 (reproduced from Digman 1990). Most psychologists consider the FFM robust (Magai and McFadden 1995), and a multi-year study found that individuals’ trait levels remained relatively stable (Soldz and Vaillant 1999). The exact names of the traits are still disputed by psychologists (Goldberg 1993; McCrae and John 1992; Digman 1990), however we adopt the common nomenclature from John and Srivastava (1999) and refer to them as:

I **E**xtraversion: How talkative, assertive and energetic a person is.
II **A**greeableness: How good natured, cooperative and trustful a person is.
III **C**onscientiousness: How orderly, responsible and dependable a person is.
IV Emotional Stability (**ES**): How calm, non-neurotic and imperturable a person is.^3^
V **O**penness to Experience: How intellectual, imaginative and independent-minded a person is.

#### Resilience

The FFM is the core model of personality, as it is considered to be stable (i.e. a person’s personality does not change, or changes very slowly). However, people also have traits that vary more quickly, encapsulate several core traits or are more environment/experience–dependent. One example is *resilience*, which is an often poorly defined term that encapsulates “the ability to bounce back from stress” (Smith et al. 2010, p. 166). Poor resilience is associated with depression (O’Rourke et al. 2010; Southwick and Charney 2012; Hjemdal et al. 2011) and anxiety (Connor and Davidson 2003; Hjemdal et al. 2011). While not as stable as the FFM traits, resilience is a medium-term trait that may be improved by interventions (Smith et al. 2010).

#### Social learning approaches

The Social Learning approach to personality “embodies the idea that both the consequences and behaviour and an individual’s beliefs about those consequences determine personality” (Carlson et al. 2004, p. 593). Whereas trait theorists argue that knowing the stable characteristics of individuals can predict behaviour in certain situations; advocates of the Social Learning approach think that the environment surrounding an individual is more important when predicting behaviours (Carlson et al. 2004). Two popular Social Learning models are Locus of Control (Rotter 1966) (LoC) and (generalized) Self-Efficacy (Bandura 1994) (GSE).

An individual’s Locus of Control represents the extent to which a person believes they can control events that affect them (Rotter 1966). A learner with an internal LoC believes that they can control their own fate, e.g. they feel responsible for the grades they achieve. A learner with external LoC believes that their fate is determined by external forces e.g. they believe that their grade is a result of the difficulty of the exam or their teaching quality. Self-Efficacy is defined as “the belief in one’s capabilities to organize and execute the courses of action require to manage prospective situations” (Bandura 1995, p. 2) and determines whether individuals will adapt their behaviour to make changes in their environment, based on an evaluation of their competency (Carlson et al. 2004). It also defines whether an individual will maintain that change in behaviour in the face of adversity; GSE has been shown to be an excellent indicator of motivation (McQuiggan et al. 2008).

### Measuring personality

There are many explicit or implicit approaches for measuring personality. Explicitly, personality traits can be obtained through *self-reporting questionnaires*, which typically ask users to rate to what extent certain statements apply to them. Multiple versions of such questionnaires exist—for example, the Five-Factor model (FFM) is often used in research, not only because there is broad agreement between psychologists, but because many validated questionnaires exist which measure it, with varying item numbers (e.g. 5 item FIPI (Gosling et al. 2003), 10 item TIPI (Gosling et al. 2003), BFI-10 (Rammstedt and John 2007), 20-item mini-IPIP (Donnellan et al. 2006), 40-item minimarkers (Saucier 1994a), 44-item BFI (John and Srivastava 1999), 50 item IPIP-NEO-50 (Goldberg et al. 2006), 60 item NEO-FFI (McCrae and Costa 2004), 240 item IPIP-PI-R, and 300-item IPIP-NEO Goldberg et al. 2006). Questionnaires for other traits also exist (see Table 2 for questionnaires that have been used for other traits). Advantages of measuring personality from self-reporting questionnaires include the ease of administration, the existence of validated questionnaires for most traits (so, easily extended to other traits), and transparency to users. Disadvantages are that they are often time consuming (leading to problems such as *straight-lining*Zhang and Conrad 2014) and may be inaccurate (either because respondents see themselves differently then they really are, or because they want to portray a certain image to other people).

Personality traits can be measured implicitly using machine learning techniques. Personality can be inferred from user generated content in social media, e.g. Facebook Likes (Kosinski et al. 2014; Youyou et al. 2015), language used (Park et al. 2015; Oberlander and Nowson 2006), Twitter user types (e.g. number of followers) (Quercia et al. 2011), a combination of linguistic and statistical features (e.g. puctuation, emoticons, retweets) (Celli and Rossi 2012), and structural social network properties (Bachrach et al. 2012; Quercia et al. 2012; Lepri et al. 2016). See Farnadi et al. (2016) for a comparative analysis.

**Table 2:** Examples of existing work on adapting to personality

References	Adapting	Personality traits	Personality measure
*Persuasive system*			
Kaptein et al. (2012, 2015)	Messages	Susceptability to Cialdini principles	STPSKaptein et al. (2012)
Orji et al. (2014)	Strategies	Gamertypes	BrainHex (Nacke et al. 2014)
Smith et al. (2016)	Reminders	FFM	Sliders (this paper)
Schiavo et al. (2016)	Group participation	FFM	BFI-10
de Vries et al. (2016)	Change processes	FFM	IPIP-NEO
Alkiş and Temizel (2015)	Strategies		BFI
Arteaga et al. (2010)	Game choice and messages	FFM	BFI-10
Halko and Kientz (2010)	Strategies	FFM	BFI
Hirsh et al. (2012)	Phone adverts	FFM	BFAS (DeYoung et al. 2007)
Lepri et al. (2016)	Social strategies	FFM	BFI
Chen et al. (2015)	Travel adverts	FFM (O,ES)	tweets; 20 from IPIP-NEO-50
Nov and Arazy (2013)	Rating UI	FFM (C)	2 from TIPI
Orji et al. (2017)	Strategies	FFM	BFI-10
Oyibo et al. (2017)	Message type	FFM	TIPI
Anagnostopoulou et al. (2017)	Strategies	FFM	BFI-10
de Vries et al. (2017)	Message type	FFM	IPIP-NEO-50
Nguyen et al. (2018)	Feedback,reminders	FFM	60 item Truity LLC (2018)
Ciocarlan et al. (2017)	Challenges	FFM (C,O,ES)	Portrayed
Orji et al. (2018)	Strategies	Gamertypes	Hexad (Tondello et al. 2016)
Ciocarlan et al. (2018)	Messages, Tasks	FFM	TIPI
*Intelligent tutoring system*			
Dennis et al. (2016)	Feedback	FFM	Portrayed
Okpo et al. (2016b, 2017)	Exercise selection	Self-esteem	Portrayed
Alhathli et al. (2016)	Material selection	FFM (E)	Portrayed
Conati and Maclaren (2009)	Educational hints	FFM (C,E,A,ES)	Personality test for children Graziano et al. (1997)
Robison et al. (2010)	Feedback type	FFM	NEO-PI-R Costa and McCrae (2008)
Harley et al. (2016)	Prompt, Feedback	FFM	mini-IPIP
Leontidis et al. (2011)	Pedag. Strategy	FFM	IPIP-NEO
Santos et al. (2016)	Affective rec. for language learning	FFM, GSE	GSE (Schwarzer and Jerusalem 1995), BFI
Santos et al. (2014)	Emotional support	FFM, GSE	GSE, BFI
McQuiggan et al. (2008)	Feedback	GSE	GSE
Sarsam and Al-Samarraie (2018)	Interface display	FFM	IPIP-NEO
*Recommender system*			
Hu and Pu (2011)	Cold-start rec.	FFM	TIPI
Nov et al. (2013)	Rating UI	FFM (E,ES)	TIPI
Tkalčič et al. (2011)	Cold-start rec.	FFM	IPIP-NEO-50
Tintarev et al. (2013)	Diversity	FFM (O)	Portrayed
Chen et al. (2016)	Diversity	FFM	25 items
Cantador et al. (2013)	Cross-domain rec.	FFM	IPIP-NEO
Quijano-Sanchez et al. (2010)	Group rec.	Accommodating, Competing, Collaborating, Compromising, Avoiding	TKI Thomas (2008)
Kompan and Bieliková (2014)	Group rec.	FFM (E,N), Competing, Coop.	NEO-FFI, TKI
Rawlings and Ciancarelli (1997)	Range of items, Popularity of items	FFM (O,E)	NEO-PI-R
Ferwerda et al. (2015)	Preferred choice for browsing	FFM (O,C,ES)	BFI
Appel et al. (2016)	Recommendations	Closeness, Curiosity, Adventurous	Social media (Gou et al. 2013)
Nunes (2008)	Recommendations	FFM	IPIP-NEO
Braunhofer et al. (2015)	Recommendations	FFM	FIPI
Odić et al. (2013)	Emotion Induction (e.g. in group vs alone)	FFM (A,E)	IPIP-NEO-50
Fernández-Tobías et al. (2016)	Cold-start rec.	FFM	MyPersonality (Kosinski 2012)
Wu and Chen (2015)	Recommendations	FFM	Implicit, 25-items
Nguyen et al. (2017)	Diversity, popularity, and serendipity	FFM	TIPI
Wu et al. (2018)	Diversity	FFM	BFI

Alternatively other interaction data can be used, such as measuring personality traits from gaming behaviour. For example, Cowley and Charles (2016) use features that describe game player behaviour based on the temperament theory of personality, Yee et al. (2011) measure personality from player behaviour in World of Warcraft, Wohn and Wash (2013) from spatial customisation in a city simulation game, and Koole et al. (2001) using a common resources dilemma gaming paradigm. Implicit association tests have also been used, measuring reaction times to visual stimuli associated with contrasting personality descriptors (Grumm and von Collani 2007).

Non-verbal data can also be used from speech and video, such as prosody, intonation, gaze behaviour, and gestures. For example, Polzehl (2014) details how speech features can be used. Biel and Gatica-Perez (2013) use features from video blogs such as speaking time, speaking speed, how much the person looks at the camera. Staiano et al. (2011) use speech and gaze attention features from videos of meetings. Rojas et al. (2011) use facial features.

Finally, multi modal personality recognition can also be used; for example Farnadi et al. (2014) used a combination of textual (linguistic and emotional) features extracted from transcripts of video blogs in addition to audio-video features. Similarly, Srivastava (2012) used a combination of non-verbal behaviour and lexical features.

For a more in depth review of automated personality recognition including a summary of existing studies and which personality traits were recognised see Vinciarelli and Mohammadi (2014).

Advantages of measuring personality implicitly are that it can be done unobtrusively (as long as the data used is generated naturally) and tends to have good accuracy. Disadvantages are potential privacy implications (it is important that users provide explicit consent), the need for substantial data for the underlying machine learning algorithms (so it requires time to measure the personality of new users) and the poor availability of existing datasets for other applications. Dunn et al. (2009) investigated ease of use, user satisfaction, and accuracy for three interfaces to obtain personality, one explicit one (NEO PI-R, with 240 questions) and two implicit ones (a game and an implicit association test). They concluded that an explicit way of measuring personality is better for ease of use and satisfaction.

### Portraying personality

Personality can be portrayed in many ways, often inspired by the ways in which it can be measured. Firstly, participants can be shown content generated by someone who with the personality trait we want to portray, such as a blog post, audio recording, or video. This is hard to do well, as it is difficult to avoid conveying information beyond personality. For example, facial expressions (as may be present in video recordings), speech (as present in video and audio recordings), and linguistic content (as present in text and speech) provide superfluous information about affective state (Zeng et al. 2009). Video, audio and text often also implicitly provide information about the person’s ethnicity/region of origin, age, gender, and opinions (Rao and Yarowsky 2010). Additionally, it requires finding those with exactly the personality trait required, and obtaining their permission for using content they generate for this purpose.

Secondly, participants can be shown such content, but rather than using a person with a desired personality trait, the trait is portrayed by an actor, researcher or automatically generated based on what we know influences the measurement of certain personality traits. This provides more control, as an actor can be instructed to depict only one trait at the extreme, and to try to be neutral on other variables, such as affective state. Social Psychology and Medical Education commonly use actors to depict personality traits. For example, Kulik (1983) used actors to portray extraversion (actor smiled, spoke rapidly and loudly, discussed drama, reunions with friends, lively parties) and introversion (actor spoke more hesitantly, talked about his law major, lack of spare time, interest in Jazz). Barrows (1987) describes stimulated/standardized patients as presenting the *gestalt* of the patient being simulated including their personality. The problem remains that actors also provide information about gender, age, ethnicity. Additionally, hiring good actors may be costly.

Portraying personality is also widely investigated in the Affective Computing community, particularly by virtual agents (Calvo et al. 2015). For example, Doce et al. (2010) convey the personality of game characters by the nature and strengths of emotions a character portrays, and their tendency to act in a certain manner. However, this is still difficult to do well, and again it is hard to do it in a way that only a personality trait is expressed and nothing more.

Thirdly, a person can be described explicitly by mentioning the personality trait (e.g. “John is very conscientious”) or how the person behaves or would behave in certain circumstances (e.g. “John tends to get his work done very rapidly”). For example, Luchins (1958) produced short stories to portray extraversion and introversion. These contained sentences such as “he stopped to chat with a school friend who was just coming out of the store” and “[he] waited quietly till the counterman caught his eye”. Using a single sentence with just the personality trait is easy to do, but it may not provide participants with a strong enough perception of the trait and it can easily be overlooked. Using a story solves this, but the story may not convey the intended trait.

In all of these cases, it is important that the portrayal of a personality trait is *validated* as accurately creating the impression of personality intended, and not producing additional impressions (of an unintended personality trait or attribute such as intelligence, etc). For example, Luchins (1958) actually found that participants associated many other characteristics (such as friendliness) based on his stories. Kulik (1983) found that prior conceptions about the actors influenced people’s opinions.

### Adapting to personality 

There is growing interest in personalization to personality, as seen from the UMUAI 2016 special issue on “Personality in Personalized Systems” (Tkalčič et al. 2016) and the “Emotions and Personality in Personalized Systems” (EMPIRE) workshops. Research on personalization to personality has focused mainly in three domains: Persuasive Technology, Intelligent Tutoring Systems, and Recommender Systems. Table 2 presents a non-exhaustive list of such research.

As shown in Table 2, research on personality in Persuasive Systems has mainly focused on adapting messages (motivational messages, prompts, adverts, reminders) and selecting persuasive strategies. Adaptation tends to use the Five Factor Model, though there has also been work on adapting to susceptibility to persuasion principles and gamer types.^4^ All papers cited use self-reporting questionnaires.

Research on personality in Intelligent Tutoring Systems has mainly focused on adapting feedback/emotional support, navigation (exercise and material selection) and hints/prompts. The Five Factor Model tends to be the basis for personality adaptation, though generalized self-efficacy (GSE) is also used. To assess personality, all papers cited used self-reporting questionnaires, except for Dennis et al. (2016), Okpo et al. (2016b) and Alhathli et al. (2016) who used indirect experiments in which participants made choices for a fictitious learner with a given personality.

**Table 3:** Self-report questionnaire for Generalized Self Efficacy (Schwarzer and Jerusalem 1995)

Statement	Score
I can always manage to solve difficult problems if I try hard enough	$_{-}$
If someone opposes me, I can find the means and ways to get what I want	$_{-}$
It is easy for me to stick to my aims and accomplish my goals	$_{-}$
I am confident that I could deal efficiently with unexpected events	$_{-}$
Thanks to my resourcefulness, I know how to handle unforeseen situations	$_{-}$
I can solve most problems if I invest the necessary effort	$_{-}$
I can remain calm when facing difficulties because I can rely on my coping abilities	$_{-}$
When I am confronted with a problem, I can usually find several solutions	$_{-}$
If I am in trouble, I can usually think of a solution	$_{-}$
I can usually handle whatever comes my way	$_{-}$

Scoring: 1 = Not at all true, 2 = Hardly true, 3 = Moderately true, 4 = Exactly true

Research on personality in Recommender Systems (see also Tkalčič and Chen 2015) has broadly considered the following topics: improving recommendation accuracy (Wu and Chen 2015), boot-strapping preferences for new users (Hu and Pu 2011; Tkalčič et al. 2011; Fernández-Tobías et al. 2016), the impact of personality on users’ preferences on recommendation *diversity* (Tintarev et al. 2013; Chen et al. 2016; Nguyen et al. 2017), cross-domain recommendation (Cantador et al. 2013), and group recommender systems (Kompan and Bieliková 2014; Quijano-Sanchez et al. 2010; Rawlings and Ciancarelli 1997). Adaptation in recommender systems aimed at individuals tends to use the FFM. However, for group recommender systems other personality traits have been used (see also Masthoff 2015) such as cooperativeness. To assess personality all papers cited used self-reporting questionnaires, except Appel et al. (2016) who extracted personality from social media usage.

## Creation of stories to express personality traits

This section describes the creation process of personality stories to express GSE, Resilience and the Five-Factor Model traits.^5^ These stories will be validated and amended in the next section. Male names were used for all stories to keep gender constant. If “gender neutral” names had been used, then participants’ interpretation of the learner’s sex may have caused an unwanted interaction effect on the validation.

### Stories for generalized self-efficacy

The self-report questionnaire for Generalized Self Efficacy Schwarzer and Jerusalem (1995) was used as a starting point, shown in Table 3.^6^ Each questionnaire item is a positively weighted value. The overall score for GSE is the sum of each scale item, with a high score (max 40) indicating high GSE.

For the high GSE story, a selection of the questionnaire items were used and changed into the third person. For the low GSE story, the valence of the items was inverted. The stories were made more realistic by associating them with a character, a first year learner called “James” (the most popular male name in English in 2010, and therefore suitably generic). The resulting stories are shown in Table 4.

**Table 4:** Stories used for Generalized Self-Efficacy, high and low

Level	Story
Low	James is a first year student. When he is faced with a difficult task, which requires him to solve a problem which he has not seen before, he tends to panic and give up, believing that he will never solve the problem. He finds it difficult to defend his ideas when someone disagrees with him. He believes that he cannot solve problems by himself. He finds it difficult to stick to his aims when learning. He tends to be quite nervous, and doesn’t believe he can pass
High	James is a first year student. When he is faced with a difficult task, which requires him to solve a problem that he has not seen before, he remains calm and believes he can always find a solution to the problem, if he tries hard enough. He believes he can defend his ideas if someone disagrees with him. He believes that he can solve any problem, whatever it is. He finds it easy to stick to his aims when learning. He is laid back about his work and believes that he will pass

### Stories for resilience

For Resilience, questions were used from the Connor-Davidson Resilience scale (Connor and Davidson 2003). These encapsulate 5 factors that contribute to resilience—Positive attitudes to change and strong relationships; Personal competency and tenacity; Spiritual beliefs and superstitions; Instincts and tolerance of negative emotions; and Control. Using questions from each factor, a story was composed for both high and low resilience (see Table 5) that are roughly symmetrical in order and content. The clauses ‘David is kind and generous’ (for both high and low stories) and ‘He is friendly’(in the low story) were added to counter the fact that the low resilience story depicted a fairly negative character.

**Table 5:** High and low resilience personality stories

Level	Story
Low	David is kind and generous. He is pessimistic and dislikes challenges. He doesn’t expect things to get better when times are tough. He gives up easily. He doesn’t believe that doing good things brings you good luck and thinks that events are down to chance. He finds it hard to deal with hardships and can’t see the positive side of tricky situations. He doesn’t feel in control of his life. He is friendly, but has few strong friendships. He is modest of his achievements
High	David is kind and generous. He is optimistic and likes challenges. He believes that when things go badly, they will always get better and he will come out stronger; whenever he fails, he tries harder until he succeeds. He tries to do the right thing because ‘what goes around comes around’. He can tough out hardships and make light of them. He feels in control of his life. He has many close friends and is proud of his successes

**Table 6:** Story construction for low emotional stability using the NEO-IPIP low items

NEO-IPIP Phrases	“Often feel blue.” “Dislike myself.” “Am often down in the dumps.” “Have frequent mood swings.” “Panic easily.” “Am filled with doubts about things.” “Feel threatened easily.” “Get stressed out easily.” “Fear for the worst.” “Worry about things”
Generated story	“Josh often feels sad, and dislikes the way he is. He is often down in the dumps and suffers from frequent mood swings. He is often filled with doubts about things and is easily threatened. He gets stressed out easily, fearing the worst. He panics easily and worries about things”

### Stories for the five factor model

Unlike GSE and Resilience, the Five Factor Personality Trait Model does not describe a single trait. As discussed in Sect. 2.1.1, the five factors (traits) are Extraversion, Agreeableness, Conscientiousness, Emotional Stability and Openness to Experience. Thus, the personality of any individual can be described by five scores, one for each of the factors. This means that stories had to be created for each trait, at both low and high level (totalling 10 stories).

To make the FFM Stories, we used the NEO-IPIP 20-item scales (Gow et al. 2005): combining the phrases into sentences to form a short story, with the addition of a name picked from the most common male names. Unlike the GSE scale, these scales provided both positive and negative items, so the high and low story could be made from the positive and negative items respectively. Table 6 exemplifies how the stories were constructed. Table 7 shows the stories.

**Table 7:** Preliminary Stories expressing each FFM trait at high and low levels

*Extraversion*	
Low	Jack has little to say to others, preferring to stay in the background. He would describe his life experiences as somewhat dull. He doesn’t like drawing attention to himself, and doesn’t talk a lot. He avoids contact with others and is hard to get to know. He retreats from others, finding it difficult to approach them. He keeps people at a distance
High	Jack feels comfortable around people and makes friends easily. He is skilled in handling social situations, and is the life and soul of the party. He knows how to start conversations and easily captivates his audience. He warms up quickly to others, and likes talking to a lot of different people at parties. He doesn’t mind being the centre of attention and cheers people up
*Agreeableness*	
Low	Charlie has a sharp tongue and cuts others to pieces. He suspects hidden motives in people. He holds grudges and gets back at others. He insults and contradicts people, believing he is better than them. He makes demands on others, and is out for his own personal gain
High	Charlie has a good word for everyone, believing that they have good intentions. He respects others and accepts people as they are. He makes people feel at ease. He is concerned about others, and trusts what they say. He sympathizes with others’ feelings, and treats everyone equally. He is easy to satisfy
*Conscientiousness*	
Low	Alexander procrastinates and wastes his time. He finds it difficult to get down to work. He does just enough work to get by and often doesn’t see things through, leaving them unfinished. He shirks his duties and messes things up. He doesn’t put his mind on the task at hand and needs a push to get started
High	Alexander is always prepared. He gets tasks done right away, paying attention to detail. He makes plans and sticks to them and carries them out. He completes tasks successfully, doing things according to a plan. He is exacting in his work; he finishes what he starts
*Emotional stability*	
Low	Josh often feels sad, and dislikes the way he is. He is often down in the dumps and suffers from frequent mood swings. He is often filled with doubts about things and is easily threatened. He gets stressed out easily, fearing the worst. He panics easily and worries about things
High	Josh seldom feels sad and is comfortable with himself. He rarely gets irritated, is not easily bothered by things and he is relaxed most of the time. He is not easily frustrated and seldom gets angry with himself. He remains calm under pressure and rarely loses his composure
*Openness to experience*	
Low	Oliver is not interested in abstract ideas, as he has difficulty understanding them. He does not like art, and dislikes going to art galleries. He avoids philosophical discussions. He tends to vote for conservative political candidates. He does not like poetry and rarely looks for a deeper meaning in things. He believes that too much tax money goes to supporting artists. He is not interested in theoretical discussions
High	Oliver believes in the importance of art and has a vivid imagination. He tends to vote for liberal political candidates. He likes to carry the conversation to a higher level, enjoying hearing new ideas. He enjoys thinking about things and can express himself beautifully. He enjoys wild flights of fantasy, getting excited by new ideas. He has a rich vocabulary

## Validation of stories to express personality traits

This section describes the validation process of each story: how each story was checked that it correctly depicted the trait that it was intended to depict (the *target trait*).

A series of validation studies were performed for the stories constructed to convey Generalised Self-Efficacy, Resilience, and the traits from the FFM (Extraversion, Agreeableness, Conscientiousness, Emotional Stability and Openness to Experience). Each trait had two stories associated with it—one to express the trait at a high level, and one to express the trait at a low level.

For each trait, at least one validation experiment was conducted (the traits from the Five Factor Model required more, this is explained further in Sect. 4.3). Each validation experiment utilized a between-subjects design: participants were shown either the high story or the low story, and then asked to rate the personality of the person depicted in the story using a validated questionnaire for the trait in question.

As outlined in Sect. 3, the stories were originally constructed using an existing personality measurement questionnaire. For validation purposes, a different measurement questionnaire was used for the same trait, as this used different language and terms to the story (preventing participants from just recognising phrases), and made the purpose of the experiment less obvious and decrease demand characteristics.

For the GSE and FFM stories, we also measured how the stories conveyed other traits (*non-target traits*), to check how they were conveyed. For GSE, we investigated how the stories conveyed the FFM traits and Locus of Control.^7^ It has been shown previously (Judge et al. 2002; Hartman and Betz 2007) that GSE interacts with both of these measures, however, if we found an unexpected interaction this would allow us to correct the story. For the FFM stories we checked how the other four non-target FFM traits were conveyed.^8^ For Resilience, which again used crowd sourcing, a different approach was taken, which is elaborated on in Sect. 4.2.

### Generalized self-efficacy (GSE) validation

This experiment explored whether stories did correctly convey different levels of GSE, and what other personality traits were implied, using a different validated trait assessment questionnaire for GSE (Chen et al. 2001). We also explored how the story depicted other traits in the FFM (using minimarkers Saucier 1994a) and a questionnaire for Locus of control (Goolkasian 2009). Fifty participants (42% female, 52% male, 6% preferred not to say; 34% aged 18–25, 48% aged 26–40, 14% aged 41–65, 2% aged over 65, 2% preferred not to say) recruited through convenience sampling in a between-subject design, answered these questionnaires, after reading the GSE personality story. 26 viewed the low GSE story and 24 viewed the high GSE story.

**Table 8:** Results of *t* tests for GSE story validation

Trait	Low GSE story		High GSE story		*p*
	Mean	SD	Mean	SD	
GSE$^{\mathrm{a}}$	**15.42**	**4.18**	**32.13**	**4.56**	$< 0.001$
Extraversion$^{\mathrm{b}}$	4.75	0.61	5.08	0.52	$> 0.05$
Agreeableness$^{\mathrm{b}}$	4.63	0.89	4.79	0.58	$> 0.05$
Conscientiousness$^{\mathrm{b}}$	**4.58**	**0.57**	**5.08**	**0.52**	$<0.05$
Emotional Stability$^{\mathrm{b}}$	4.93	0.73	4.71	0.97	$> 0.05$
Openness$^{\mathrm{b}}$	4.82	0.68	4.83	0.46	$> 0.05$
Locus of Control $^{\mathrm{c}}$	**8.92**	**2.76**	**2.67**	**2.28**	$<0.001$

Bold values indicate significant difference between high and low story

$^{\mathrm{a}}$From 8 to 40 with 8 lowest

$^{\mathrm{b}}$From 1 to 9 with 1 lowest

$^{\mathrm{c}}$From 0 to 13 with 0 indicating entirely internal locus and 13 indicating entirely external locus

Table 8 shows the results. *t* tests^9^ were run for each of the traits to test whether the high and low GSE stories were significantly different from each other. This was significant at $t(48)=-\,13.514$, $p<0.001$. A Point-Biserial Correlation showed a significant difference ($r(50)=0.89$, $p<0.001$, $R^2=0.79$), showing a strong effect size for the GSE Stories.

The stories did however express some other personality traits and models at significantly different levels (Conscientiousness and Locus of control). However, this was to be expected as GSE is not an isolated construct: previous research has discussed possible correlations between GSE and other psychological constructs, including conscientiousness and locus of control (Judge et al. 2002; Hartman and Betz 2007). We therefore judged that these stories were sufficient for further experiments.

### Resilience validation

Similarly to GSE, resilience is expected to correlate with other personality traits. We validated that the high and low stories depicted high and low resilience; no other traits were compared as it was anticipated that there would be an interaction (e.g. with low emotional stability) and this is not a problem for this measure. 44 participants were recruited through MT (26 female, 17 male, 1 undisclosed, aged 18–65). They were shown either the high or low story (between-subjects design) and asked them to assess the person in the story on the six item ‘Brief Resilience Scale’ (Smith et al. 2008). We added six items from another scale to mitigate hypothesis guessing and reduce response bias.

To validate the stories, we performed a between-subjects *t* test to test Average Resilience rating between the low and high stories. This was significant at $t(41)=0.29$, $p<0.001$. The mean resilience rating was 1.75 ± 0.51 SD for the low story and 4.20 ± 0.49 SD for the high story on a 1–5 scale. A Point-Biserial Correlation showed a significant difference ($r(43)=0.93$, $p<0.001$, $R^2=0.85$), showing a strong effect size for the Resilience Stories.

### Five factor trait validation

This section is an improved version of previous research reported in Dennis et al. (2012b), with clarifications and an additional effect size analysis.

**Fig. 2:**
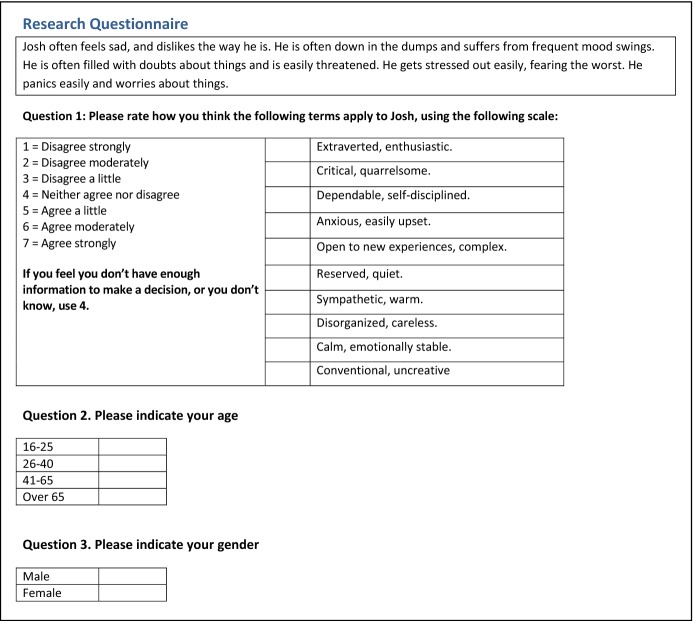
The pilot story validation questionnaire, for Emotional Stability

#### First iteration FFM: pilot study

The Emotional Stability stories from the FFM were used for a validation pilot study for the FFM traits, and to determine whether non-target trait mitigation would be required.

The same methodology from Sect. 4.1 was used. Eight participants (4 female; 5 aged 18–25, 3 aged 26–40) recruited through convenience sampling (4 students and 4 staff at the University of Aberdeen) were presented with one of the stories using a between-subjects design and asked to judge them on personality. However, as this was a pilot study, instead of using the 40 item minimarkers to judge the FFM, we used a TIPI questionnaire (Gosling et al. 2003) with 10 items instead (for brevity), shown in Fig. 2. The results are shown in Table 9.

**Table 9:**
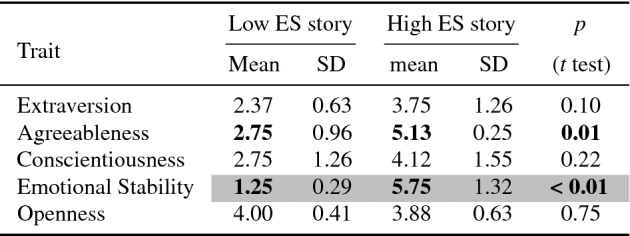
Results of pilot study for ES stories (high and low), as rated using TIPI for the FFM traits

Values could range between 1 and 7. Bold values indicate significant difference between high and low stories. Grey cells indicate trait designed to convey

The stories did convey Emotional Stability at polarized levels (i.e. the ratings for each story were at opposite ends of the scale for ES). However, there appeared to be a positive correlation with Agreeableness—more emotionally stable people were judged to be more agreeable (nicer) than neurotic ones. This effect could be spurious due to the low number of participants, or due to our decision to use the ten-item TIPI test rather than a more comprehensive test with a higher number of items. For more formal validation, a large number of unique participants is required for reliable data, particularly if adjustments to the stories are required. The second iteration uses a larger set of participants recruited through crowd-sourcing to establish whether the correlation with Agreeableness persists and also attempts to validate the stories for the other FFM traits.

#### Second iteration: validation of stories for the five factor model

100 participants (10 per story; 67% female) were recruited using MT. In a between-subjects design, each participant was presented with one story about a learner (see Table 7) which attempted to convey a target trait at either a high or low-level. Participants assessed this student’s personality using the Mini-Markers scale (Saucier 1994a).

**Table 10:** Normative ranges for each of the five traits, arising from the ratings of a liked peer for the minimarkers scale (Saucier 1994b), plus or minus one standard deviation

Trait	E	A	C	ES	O
Normative range	4.75–7.63	5.10–7.96	4.48–7.10	3.72–6.08	4.99–7.45

The rating for the target trait (i.e. the trait that the story was created to express) should be as polarized as possible—the “low” variant of a story aimed for a score as close to 1 as possible, and the “high” story aimed for a score as close to 9 as possible.

The decision for an acceptable value for a non-target trait is rather arbitrary. However, it is possible to derive normative values for each trait from large population samples. As these samples are similar to our own (e.g. English-speaking, USA-based), we decided it was acceptable to use these to characterise people as being either ‘high’, ‘low’ or ‘neutral’ in a trait.

To decide on acceptable values for non-target traits, a “normative range” was made for each of the five traits based on the average ratings of a liked peer for the minimarkers scales from 329 students from Illinois (Saucier 1994b),^10^ plus or minus one standard deviation, shown in Table 10.

*Results* Table 11 shows the results of the original stories. There was a significant difference between all 5 pairs of stories in the perceived trait values for the target trait between the high story and the low story. For all but one personality trait (Openness), the perceived target trait values were clearly outside the normative range and in the correct direction. The perceived target trait value for low openness is below the normative range, but high story marginally outside the normative range. Problematically, there were many significant differences between the perceived non target trait values. Several perceived non-target trait values were also outside the normative range.

**Table 11:**
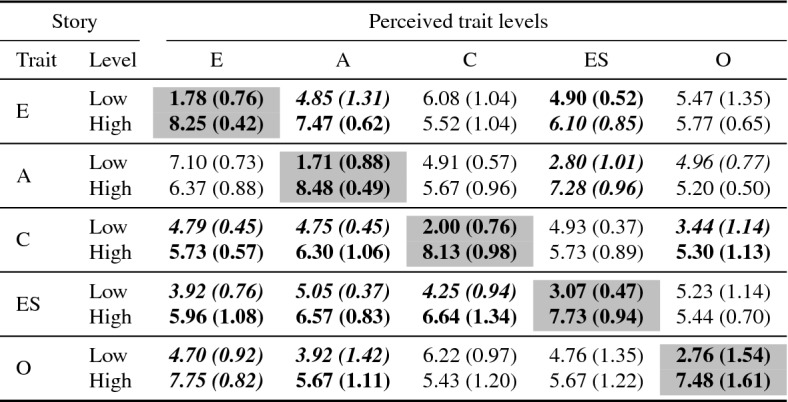
Results for FFM stories

Bold items indicate $p < 0.05$, (*t* test Bonferroni corrected) between low/high stories. Grey cells indicate target trait levels. Italics indicate non-target trait outside normative range. Target trait score underline—score not outside normative range

#### Mitigation

The following problems occurred between the pairs of stories during validation:

1. Perceived trait values on a non-target trait differ significantly
2. Perceived trait values on a non-target trait are outside the normative range
3. Perceived target trait values are very close to normative range

Problems P1 and P2 often appeared together—one (or both) of the perceived values for a non-target trait were outside the normative range and thus significantly different from the other. For example, in the story for low extraversion, the student was perceived to be less agreeable, despite correctly conveying low extraversion and the scores for the remaining non target traits being within the normative range. We hypothesised that the following story modifications could be taken in an attempt to mitigate problems P1 and P2:

1. Add a statement which implies a semi neutral stance on the problem trait, e.g. “Jack is quite a nice person” to mitigate low agreeableness.
2. Remove a statement which may be causing the interaction—e.g. removing “Jack has little to say to others” may increase agreeableness.
3. Add a statement targeting the problematic non-target trait from its own story—e.g. adding “Jack has a good word for everyone” from the high agreeableness story to increase agreeableness in other stories.

S1 was used because S2 (removing statements from the stories) was undesirable: this may affect the story’s expression of the target trait. We did not attempt S3 as it may over-alter the non-target trait score, and introducing another trait into a story may bring that trait’s undesirable interactions into the story. For example, the low conscientiousness story also conveys low agreeableness (see Table 16). If we added a statement from the high agreeableness story, this could in turn raise the ES score, as the high agreeableness story also conveyed high ES (further confounding the problem).

**Table 12:** Mitigating Statements for each non-target FFM trait

Non-target trait	Statement to add if below normative
Extraversion	Tends to enjoy talking with people
Agreeableness	Quite a nice person
Conscientiousness	Tends to do his work
Emotional stability	Tends be calm
Openness	Quite likes exploring new ideas

**Table 13:** Two stories for high Openness to Experience

Original story	Oliver believes in the importance of art and has a vivid imagination. He tends to vote for liberal political candidates. He likes to carry the conversation to a higher level, enjoying hearing new ideas. He enjoys thinking about things and can express himself beautifully. He enjoys wild flights of fantasy, getting excited by new ideas. He has a rich vocabulary
Modified story	Oliver believes in the importance of art and has a vivid imagination. He tends to vote for liberal political candidates. He enjoys hearing new ideas and thinking about things. He enjoys wild flights of fantasy, getting excited by new ideas

#### Third iteration: validation with mitigated sentences

As the undesired non-target trait scores occurred most frequently in the low stories, these were targeted first. We constructed slightly positive statements (see Table 12) and added them where necessary. For the ‘high’ stories, only two non-target traits required modification: Extraversion in the Openness High story, and Emotional Stability in the Extraversion High and Agreeableness High stories. For the Extraversion High story, the score for Emotional Stability was 6.10, and the normative range ends at 6.08. Because this margin was so small, and there was no significant difference between the high and low variants’ ES scores, modification was not attempted to avoid more adverse effects. In the case of the high Agreeableness story, the value for ES was 7.28. S1 was employed by adding a mildly negative statement: “He is occasionally a bit anxious”. The Openness High story did not convey its target trait convincingly, and thus already required modification. Approach S2 was used in this case, removing statements such as “[he can] express himself beautifully” (see Table 13).

*Design* The design was the same as Sect. 4.3.2. Seventy participants (10 per adjusted story) were recruited from MT. Each participant saw one story in a between-subjects design.

*Results* Tables 14 and 15 shows the results for the modified stories. S1 was successful in most cases in mitigating P1 and P2. Exceptions to this were in the Agreeableness stories, the undesired non-target trait scores still remain, with the Low story expressing low ES and the High story expressing high ES (P1 and P2). For Conscientiousness, P1 occurred for Openness, despite both values being in the normative range. For low Emotional Stability, S1 was not effective for bringing the perceived trait value into normative range for Extraversion, with P1 and P2 still extant. S2 was successful in solving P2 for Openness High; bringing the Agreeableness value into the normative range. However, we were not successful in solving P3 for Openness high; the score for the target trait is further within the normative range.

*Effect Size for Modified Stories* To explore how strongly the high and low stories differed for each trait, a Point-Biserial correlation was computed between the high and low stories for each trait. There was a strong positive correlation between the story trait level (low or high) and trait score for each trait, showing that the stories depict the traits strongly at the intended levels (see Table 14).

**Table 14:** Point-Biserial correlations between the high and low story for each trait

Trait	*r*	$R^2$	*p*
Agreeableness	0.95	0.90	$<0.001$
Extraversion	0.99	0.97	$<0.001$
Openness to Experience	0.87	0.76	$<0.001$
Conscientiousness	0.98	0.95	$<0.001$
Emotional stability	0.95	0.89	$<0.001$

**Table 15:**
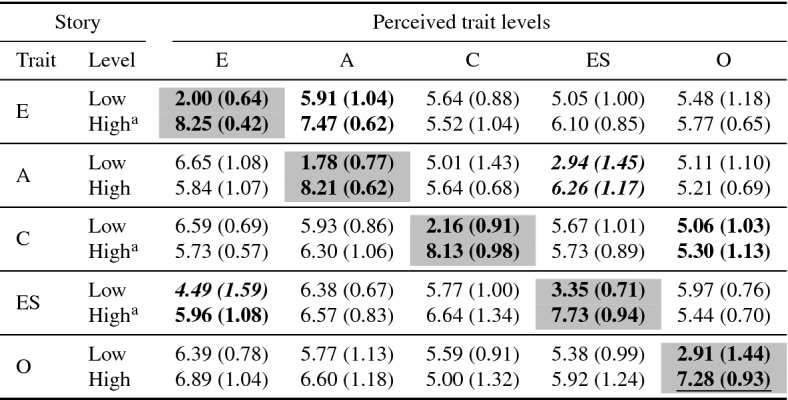
Results for corrected FFM stories

Bold items indicate $p< 0.05$, (*t* test Bonferroni corrected) between low/high stories. Grey cells indicate target trait levels. Italics indicate non-target trait outside normative range. Target trait score underline—score not outside normal range

$^\mathrm{a}$Story not adjusted, previous values used

**Table 16:** Validated stories for each FFM trait, high and low

*Extraversion*	
Low	Jack has little to say to others, preferring to stay in the background. He would describe his life experiences as somewhat dull. He doesn’t like drawing attention to himself, and doesn’t talk a lot. He avoids contact with others and is hard to get to know. He retreats from others, finding it difficult to approach them. He keeps people at a distance. Jack is quite a nice person
High	Jack feels comfortable around people and makes friends easily. He is skilled in handling social situations, and is the life and soul of the party. He knows how to start conversations and easily captivates his audience. He warms up quickly to others, and likes talking to a lot of different people at parties. He doesn’t mind being the centre of attention and cheers people up. Jack can sometimes be insensitive
*Agreeableness*	
Low	Charlie has a sharp tongue and cuts others to pieces. He suspects hidden motives in people. He holds grudges and gets back at others. He insults and contradicts people, believing he is better than them. He makes demands on others, and is out for his own personal gain. Charlie tends to be calm and quite likes exploring new ideas
High	Charlie has a good word for everyone, believing that they have good intentions. He respects others and accepts people as they are. He makes people feel at ease. He is concerned about others, and trusts what they say. He sympathizes with others’ feelings, and treats everyone equally. He is easy to satisfy. Charlie tends to be quite anxious
*Conscientiousness*	
Low	Josh procrastinates and wastes his time. He finds it difficult to get down to work. He does just enough work to get by and often doesn’t see things through, leaving them unfinished. He shirks his duties and messes things up. He doesn’t put his mind on the task at hand and needs a push to get started. Josh tends to enjoy talking with people
High	Josh is always prepared. He gets tasks done right away, paying attention to detail. He makes plans and sticks to them and carries them out. He completes tasks successfully, doing things according to a plan. He is exacting in his work; he finishes what he starts. Josh is quite a nice person, tends to enjoy talking with people, and quite likes exploring new ideas
*Emotional stability*	
Low	James often feels sad, and dislikes the way he is. He is often down in the dumps and suffers from frequent mood swings. He is often filled with doubts about things and is easily threatened. He gets stressed out easily, fearing the worst. He panics easily and worries about things. James is quite a nice person who tends to enjoy talking with people and tends to do his work
High	James seldom feels sad and is comfortable with himself. He rarely gets irritated, is not easily bothered by things and he is relaxed most of the time. He is not easily frustrated and seldom gets angry with himself. He remains calm under pressure and rarely loses his composure
*Openness to experience*	
Low	Oliver is not interested in abstract ideas, as he has difficulty understanding them. He does not like art, and dislikes going to art galleries. He avoids philosophical discussions. He tends to vote for conservative political candidates. He does not like poetry and rarely looks for a deeper meaning in things. He believes that too much tax money goes to supporting artists. He is not interested in theoretical discussions. Oliver is quite a nice person, and tends to enjoy talking with people
High	Oliver believes in the importance of art and has a vivid imagination. He tends to vote for liberal political candidates. He enjoys hearing new ideas and thinking about things. He enjoys wild flights of fantasy, getting excited by new ideas

#### Discussion

The adjusted FFM stories are shown in Table 16. A story expressing a single polarized trait was always going to be difficult to achieve as the traits within the FFM are intercorrelated (Chamorro-Premuzic 2011). The interaction between Agreeableness and Emotional Stability was too strong to remove entirely. Adding a stronger statement to bring Emotional Stability into the normal range may cause more interactions with the other three non-target traits. In the Conscientiousness and Extraversion stories—the score for certain non target traits (O and A, respectively) still significantly differed. However, as these were all in the normal range, we do not see this as a problem. Problem P3 was not solved in the case of High Openness. Openness is a difficult trait to conceptualise—incorporating culture and art as well as political beliefs (Chamorro-Premuzic 2011). The perceived score was high, so it is likely therefore that it was expressing Openness highly, just not outside the range we devised.

### Conclusion and limitations

A set of stories for the FFM, GSE and Resilience have been constructed and validated. Not all FFM stories are perfect, modifying them seemed to “dilute” the effect of the target trait, implying a balancing act. Further strategies could be used to remove the remaining interactions, however it may be that one trait inevitably infers another. We judge that the stories are good enough at expressing the traits for the purpose of investigating adaptation to personality in intelligent systems.

## Using stories to determine personality

In this section we investigate how to use the stories to measure personality. Participants were given a standardised personality test and asked to rate how close they were to a pair of diametrically opposed personality stories using a sliding scale. A correlational analysis was performed on each trait to show that the sliding scale measured the trait with a strong correlation coefficient. We then conducted a reliability check, where a new sample of participants completed the sliders twice, 1 week apart. The scores between week 0 and week 1 were strongly correlated—thus the sliders could be used to measure personality (though this should not replace a standardised test when high granularity is required).

### Methods

#### Materials

**Fig. 3:**
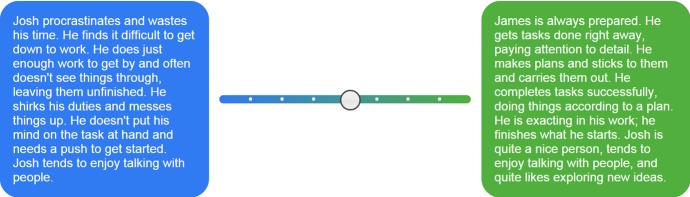
Screenshot of the slider between opposing trait stories

The validated stories were taken from Tables 4, 5 and 16. Different common Western names were used for each story, gender-matched to the participant. These were formatted so that opposing stories of the same trait were placed at either end of a sliding scale (see Fig. 3). The scale was coloured using a gradient from blue to green (left to right), with markers every 12.5%. The participant could indicate their position on the scale using a drag-and-drop slider. The position of the positive and negative stories was randomised for each participant and for each trait. The slider position gave a value of between 18 and 162, emulating a conventional 1–9 scale with greater acuity.

Validated personality questionnaires were used. For the Five Factor Model, the minimarker test (Saucier 1994a) was used. For resilience, the Brief Resilience Scale was used (Smith et al. 2008). For self-efficacy, the general self-efficacy scale was used (Schwarzer and Jerusalem 1995).

#### Procedure

Participants completed a personality questionnaire and then were presented with the slider test for each trait of the personality questionnaire they had completed, one at a time (five pairs of sliders for the Big Five Minimarker questionnaire and one pair of sliders for each other questionnaire).^11^ Participants were asked to move the slider towards the person they thought they were most like. The slider was initially set at the 50% marker on the scale and participants had to manipulate the slider before they were allowed to continue, even if they chose to select 50%. Participants were then thanked for their time and invited to view the results of the slider test in the form of a bar graph. Participants were recruited from MT and were paid $0.80 (demographics shown in Table 17).

**Table 17:** Participant demographics for FFM, Self Efficacy and Resilience for slider validation studies

Story set	Age					Gender			Total
	18–25	26–40	41–65	Over 65	n.d.	F	M	Other	
FFM	14	33	13	1	0	29	32	0	61
Self Efficacy	10	33	18	1	0	25	37	0	62
Resilience	13	31	15	1	0	19	41	0	60

#### Design

Participants completed both the personality questionnaire and the slider test in a within-subjects design. Their score on the personality questionnaire was the independent variable and the Value of the slider position (which represents how close to the 2 trait stories the participant thought they were) was the dependent variable.

Our hypothesis (H1) was: For each trait, there will be a positive correlation between personality score and slider value.

### Results

#### Five factor model

**Fig. 4:**
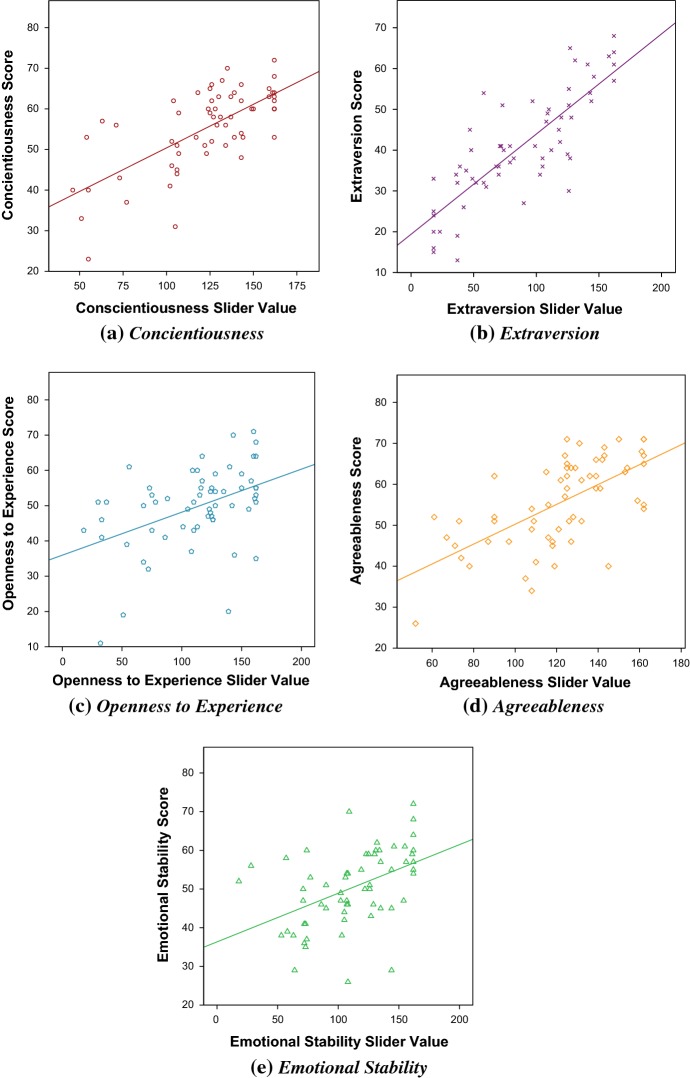
Correlation of Trait Score $\times$ Slider Values for the FFM personality traits

For each trait, a correlation analysis was run of Trait Score $\times$ Slider Value. This was significant for each trait (see Table 18). Correlation graphs were plotted for each trait (Fig. 4) and a regression analysis run. The regression formula for each trait is shown in Table 18. Participants’ mean scores on the minimarkers scale (see Table 19) were compared with the minimarkers normal range (see Table 10) to see if the MT participants’ varied from a normal population. All traits were within the normal range, except emotional stability which was slightly higher. To investigate the effect of other traits on the correlation for each trait, a partial correlation analysis was run to control for the effect of non-target traits. This correlations remain strong (see Table 20).

**Table 18:** Pearson’s *r* for correlation of Trait Score $\times$ Slider Value for each personality trait, effect size $R^2$, regression formula and standardized error of the estimate *SEE*

Trait	*r*	$R^2$	*p*	*n*	Regression formula for slider	*SEE*
Conscientiousness	0.69	0.48	$<0.01$	61	2.23 $\times$ ConScore − 0.22	23.46
Extraversion	0.82	0.67	$<0.01$	61	2.71 $\times$ ExtScore − 23.56	25.90
Openness to Experience	0.44	0.19	$<0.01$	61	1.58 $\times$ OpExScore + 33.39	37.19
Agreeableness	0.64	0.41	$<0.01$	61	1.67 $\times$ AgrScore + 29.48	21.60
Emotional Stability	0.46	0.21	$<0.01$	61	1.67 $\times$ EmStScore + 27.16	32.64
Resilience	0.58	0.37	$<0.01$	60	3.39 $\times$ ResScore + 43.25	25.53
GSE	0.62	0.38	$<0.01$	62	3.33 $\times$ GseScore + 26.54	19.93

#### Resilience and generalised self efficacy

**Fig. 5:**
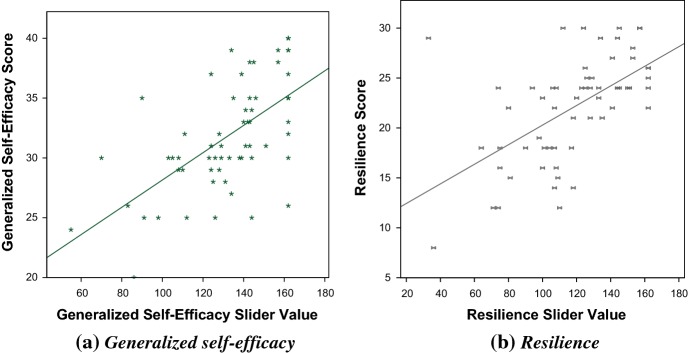
Correlation of Trait Score $\times$ Slider Value for GSE and Resilience

For each personality test, correlation graphs were plotted (Fig. 5) and a correlation analysis was run of Test Score $$\times $$ Slider Value. This was significant for Resilience ($$r(60)=0.58$$, $$ p< 0.01$$) and GSE ($$r(62)=0.62$$, $$p < 0.01$$). The regression formula for each trait is shown in Table 18.

### Reliability check

To test the reliability of the sliders, a reliability check experiment was conducted using all 7 sliders (FFM, GSE and Resilience). Participants recruited through opportunistic sampling completed the sliders and the FFM TIPI test (Gosling et al. 2003) as the first part of a persuasion experiment (reported in Ciocarlan et al. 2019). After 1 week they completed the sliders and TIPI test again (as well as the second part of the persuasion experiment).

Fifty-one participants completed the study (27 female, 23 male, 1 undisclosed; 21 aged 18–25, 23 aged 26–40, 7 aged 40–65). A correlation analysis was run between Slider Values for Week 0 $\times$ Week 1 for all traits. The results are shown in Table 21. There was a strong correlation for each of the sliders between Week 0 and Week 1 ($r=0.70$–0.86, mean $=0.81$). There were several other significant weaker correlations—expected correlations between FFM traits and GSE and Resilience (as these traits are known to correlate with FFM traits; see Section 4), and some correlation within FFM traits.

To explore the inter-trait correlations within the FFM traits, a correlational analysis was run for the TIPI test for each FFM trait between Week 0 and Week 1. The results are shown in Table 22. We found a similar pattern of correlation between non-target traits as we found in the sliders, with the TIPI test showing more correlations between non-target traits than the slider test. We can therefore see that the inter-trait correlations are captured by a validated personality test within our sample, and that the sliders show good test-retest reliability for target traits at Week 1.

Additionally, we used the data from Week 0 to repeat our validation experiment for the FFM sliders. A correlational analysis of FFM slider values $\times$ TIPI test scores showed a significant correlation between each trait’s score on the slider test and TIPI test (E: $r=0.78$, A: $r=0.62$, C: $r=0.62$, ES: $r=0.83$, O: $r=0.33$; $p<0.01$ for E, A, C and ES, $p<0.05$ for O). These are similar to correlations reported in Table 18; O has a weaker correlation and ES has a stronger correlation in this reliability check.

**Table 19:** Means of study participants for the minimarkers scale

Trait	Extraversion	Agreeableness	Conscientiousness	Emotional stability	Openness to experience
Mean	5.09	6.95	6.92	6.29	6.25

**Table 20:** Partial correlations of each FFM trait on Minimarkers compared with the slider score, controlling for each other trait score on the non-target sliders

Partial correlations	*r*	*p*
Agreeableness	0.57	$<\,0.001$
Extraversion	0.75	$<\,0.001$
Emotional Stability	0.34	0.010
Conscientiousness	0.61	$<\,0.001$
Openness to Experience	0.36	0.006

**Table 21:**
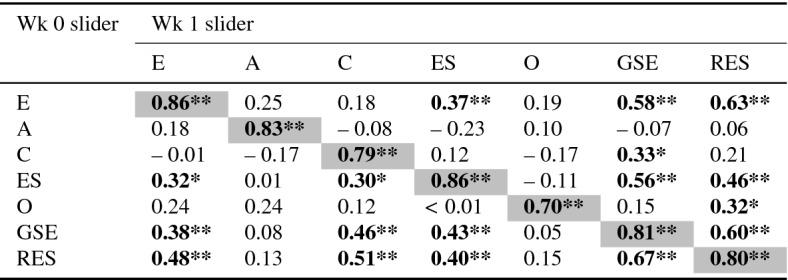
Pearson’s *r* Correlation of the slider value of each pair of stories: FFM (E, A, C, ES, O), GSE and Resilience, repeated after 1 week

Grey cells indicate the correlation of same trait at week 0 and week 1

$$^{*}p<0.05;\,\,{^{**}}p<0.01$$

**Table 22:**
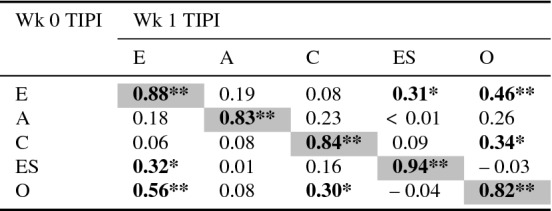
Pearson’s *r* Correlation of the FFM TIPI test score (E, A, C, ES, O) at Week 0 and Week 1

Grey cells indicate the correlation of same trait at week 0 and week 1

$$^{*}p<0.05;\,\,{^{**}}p<0.01$$

### Interpreting slider values

There are several possible strategies in the interpretation of the slider values for use in personality experiments. The slider values form a continuous variable, which can be used in analysis for further studies (e.g. using a regression analysis). Splitting data into distinct groups is often considered undesirable, as it causes the data to lose power (Irwin and McClelland 2003). However, for some studies it may be useful to use the slider values to divide participants into High and Low groups (for example, when you want to offer different content to people with different traits).

When choosing to divide participants into groups, it is important to consider statistical features of the data (e.g is the data statistically normal), as well as the purpose of the study, and the limitations of data collection. For non-normal data, data can be split using the median, tertiles or quartiles. For normal data, groups can be formed using the mean or standard deviation. A further option is to take the highest and lowest scoring participants to form a defined group size (e.g. top 50 and bottom 50), or to use a hybrid method (e.g. the top and bottom 20 participants at least 1 standard deviation from the mean). It is also possible to compute the equivalent score on a standardised test (e.g. the TIPI test), by using the regression formula generated at validation (e.g. in Table 18) and group by population normative data for that test, when available (e.g. Table 10). The choice should be guided by how much data can be discarded, the importance of groups being distinct from each other, and how many groups are required (i.e. a ‘neutral’ group required). This is summarised in Table 23.

### Discussion

This section has demonstrated how to use trait stories to measure personality. For each trait, there is a strong correlation between participants’ scores on standardised personality tests and their scores on the slider scale (see Table 18). The effect size of the correlations imply that more polar trait stories (i.e. pairs of stories that are rated as very high and low in the trait) result in a sliding scale that better reflects the personality test. This can be seen in the comparatively low correlation for the Openness to Experience slider in Table 20. This highlights the importance of the story validation stage of development.

It should be noted that, while the sliders may be preferable to questionnaires, they have a lower accuracy than many standardised questionnaires. As for any decision about which measure to use in a study, the benefits of using the slider measure should be weighed against its lower accuracy; e.g. where high attrition needs to be mitigated by simplifying the questionnaires, or where the intended analysis groups users by trait.

**Table 23:** Summary of ways to divide Personality Slider data into groups

	Regression	Median	Quartile	Tertile	Group size	Mean split	SD	Hybrid
Suitable for non-normal data	$\checkmark$	$\checkmark$	$\checkmark$	$\checkmark$				$\checkmark$
Suitable for normal data	$\checkmark$				$\checkmark$	$\checkmark$	$\checkmark$	$\checkmark$
Groups equal size		$\checkmark$	$\checkmark$ $^\mathrm{a}$	$\checkmark$	$\checkmark$			$\checkmark$
Distinct high/low groups	$\checkmark$		$\checkmark$	$\checkmark$	$\checkmark$		$\checkmark$ $^\mathrm{b}$	$\checkmark$
‘Normal’ group	$\checkmark$ $^\mathrm{c}$		$\checkmark$	$\checkmark$			$\checkmark$	$\checkmark$
No data discarded			$\checkmark$	$\checkmark$		$\checkmark$		
Groups reflect population norms	$\checkmark$							

$^\mathrm{a}$Double size normal group

$^\mathrm{b}$ Groups are statistically different from each other

$^\mathrm{c}$Only possible if high and low thresholds are defined by other research

## Applying stories and sliders in personality research and beyond

This section provides examples of how the personality stories and sliders, and the method used to produce them, have been used in adaptation research, for adaptation to personality and beyond, demonstrating evidence of the method’s usefulness.

### Portraying personality

Personality stories provide an easy way of portraying certain personalities as needed for indirect and user-as-wizard studies. Based on our research (i.e. Sect. 4), using personality stories also ensures (as far as possible) that the impression of the participant of the person’s personality is in accordance to what the story is intended to express. Personality stories have been used for investigations into adaptation in persuasive technology, intelligent tutoring systems, and recommender systems (see Table 24). In Dennis et al. (2015) an indirect study was run with 68 participants investigating the impact of a skin cancer patient’s personality on the perceived suitability of reminder messages (varied types based on Cialdini principles Cialdini 2001) to self-check their skin. Participants were provided with a personality story about a fictional skin cancer patient. They rated the suitability of reminder messages for this patient and selected the best message to use. Results showed a significant difference between participants based on levels of Conscientiousness: those high in Conscientiousness preferred authority messages as the second reminder whilst those low in Conscientiousness preferred scarcity messages.

**Table 24:** Studies using personality stories and sliders to obtain or portray personality

Use	References	Domain	Stories	Task
Portraying	Dennis et al. Dennis et al. (2015)	Persuasion	FFM (C)	Judge reminder persuasiveness
	Dennis et al. (2012a, 2013, 2016)	ITS	FFM	Provide feedback and emotional support
	Dennis et al. (2011)	ITS	GSE	Provide feedback
	Smith et al. (2015)	eHealth	FFM (ES)	Provide emotional support
	Smith (2016)	eHealth	Resilience	Provide emotional support
	Tintarev et al. (2013)	RecSys	FFM (O)	Select an item set
	Okpo et al. (2016a, b, 2018)	ITS	Self-esteem	Select exercise difficulty
Obtaining	Alhathli et al. (2016, 2017)	ITS	FFM	Judge learning materials
	Smith and Masthoff (2018)	eHealth	FFM	Judge emotional support messages
	Smith et al. (2016)	Persuasion	FFM	Judge reminder persuasiveness for a person with their own personality
	Thomas et al. (2017); Josekutty Thomas et al. (2017)	Persuasion	FFM	Judge healthy eating messages

In Dennis et al. (2016), five user-as-wizard studies were run with 1203 participants in total, each investigating the impact of one of the FFM personality traits (as well as performance) on feedback (emotional support and slant) given to a learner. Participants were provided with a personality story about a learner and their performance, and provided feedback. Based on this data, an algorithm was developed that adapted feedback to Conscientiousness and Emotional Stability.

In Dennis et al. (2011), a User-as-Wizard study was run with 19 teachers, investigating the impact of GSE on feedback (slant). Participants were provided with a GSE personality story about a learner and their performance, and produced feedback. There was some evidence of teachers putting a positive spin on feedback for learners with a low GSE.

In Okpo et al. (2017), a User-as-Wizard study was run with 201 participants, investigating the impact of the Self-Esteem personality trait (as well as effort and performance) on exercise selection (difficulty level). Personality stories were constructed for Self-Esteem using the methodology presented in this paper. Participants were provided with either a low or high self-esteem story, the effort put in by the learner and their performance on a previous exercise. Participants selected the difficulty level of the next exercise for the learner to do. Self-esteem had an impact on difficulty level selection.

In Tintarev et al. (2013), a User-as-Wizard study was run with 120 participants, investigating the impact of Openness to Experience on recommendation diversity. Participants were provided with a personality story about a fictional friend as well as some indication of that friend’s book preferences, and provided three book recommendations to this friend. There was some evidence that participants took Openness to Experience into account when producing the recommendations.

In Smith et al. (2015) and Smith (2016), two User-as Wizard studies were run with 61 and 45 participants respectively, investigating whether emotional support messages should be adapted to the recipient’s Emotional Stability and Resilience respectively. Participants were provided with a personality story about a carer experiencing a stressful situation, and provided emotional support messages for this carer. Results showed that neurotic carers were provided with a wider range of emotional support. No effect was found of resilience on message selection.

### Obtaining personality

Some studies require participants’ personalities in order to analyse the impact of that personality on dependent variables (e.g. participants’ preferences, participants’ learning, etc). Most of the studies presented in Table 2 are of this type. The personality sliders have been used to obtain participants’ personality to investigate adaptation in persuasive systems and intelligent tutoring systems. See Table 24 for example studies.

In Smith and Masthoff (2018), a study was run with 138 participants investigating the impact of personality on their appreciation of emotional support messages for stressful situations. Participants were told about a carer experiencing a stressful situation and rated an emotional support message provided by the carer’s friend on how helpful, effective and sensitive they felt it was. Participants’ FFM personality traits were obtained using personality sliders. Results showed that personality only had a small impact, with agreeableness and emotional stability warranting further investigation.

In Smith et al. (2016), an indirect study was run with 51 participants investigating the impact of personality on perceived persuasiveness of reminder messages (differing in type based on Cialdini principles Cialdini 2001) to self-check their skin for skin cancer patients. Participants’ FFM traits were obtained using the personality sliders. They were told about a skin cancer patient who had the same personality as themselves and rated the suitability of reminder messages for this person. Results showed that personality is important when deciding on the type of persuasion to use in reminder messages.

In Thomas et al. (2017) and Josekutty Thomas et al. (2017), an indirect study was run with 152 participants investigating the impact of personality on the perceived persuasiveness of healthy eating messages differing in type and framing (positive or negative). Using the FFM personality sliders, the participants’ personalities were obtained. They rated the perceived persuasiveness of messages for someone with a similar personality as themselves. There was some evidence of conscientiousness impacting persuasiveness.

In Alhathli et al. (2016), an indirect study was run with 50 participants exploring the impact of a learner’s extraversion on the selection of learning materials (active vs passive, and social vs individual). Participants’ personalities were obtained using the FFM personality sliders and they were told the learner had the same personality as them. They rated learning materials on the extent they felt the learner would enjoy them and they would increase the learner’s skills and confidence. Extraversion was found to impact perceived enjoyment of social learning materials. In Alhathli et al. (2017), a similar study was run with 163 participants where the learning materials reflected learning styles, and participants’ learning styles were measured in addition to their personality. No impact of either personality or learning style was found.

Results from these studies showed that the slider results can be used both for correlation analyses and to divide participants into high/low groups on different traits.

### Applying the method beyond personality research

Finally, the method described in this paper for developing validated stories can also be applied to non-personality user or context characteristics. We have successfully applied this in multiple studies—for example, Smith et al. (2014) and Kindness (2014) developed stories that depicted different types of stressors experienced respectively by carers and community first responders. Forbes et al. (2014) developed stories that depicted different attitudes towards usage of transport means. In all of these cases, the stories were used to bootstrap adaptation research.

## Conclusion

Increasingly, as illustrated in Sect. 2.4, research on adaptive systems is investigating personality as a user characteristic for adaptation. However, to do this effectively, reliable and lightweight ways are needed to express personality (for use in indirect and user-as-wizard studies) and to obtain user-personality. The paper makes two major contributions to this.

Firstly, the paper contributes a methodology for creating and validating stories that reliably express a personality trait. To illustrate the methodology, the paper presented the creation and validation of stories expressing the Five Factor model traits (extraversion, agreeableness, conscientiousness, emotional stability, openness to experience), generalized self-efficacy, and resilience. The usefulness of the personality stories for adaptation research has been shown by the many examples provided of their use for indirect and user-as-wizard studies (see Sect. 6).

Secondly, the paper contributes a lightweight methodology for obtaining user-personality, using the personality stories as part of a self-assessment scale. These personality story scales can be used in studies investigating the impact of a trait, and may also be used by a system to allow it to adapt to this trait. The paper contributes guidelines on how to use such scales. The usefulness of the personality story scales for obtaining study participants’ personality has been shown by their usage in adaptation studies (see Sect. 6).

While this paper looks at a small number of personality traits, the methodology can be extended to any user factor for which a validated questionnaire exists. So, as indicated in Sect. 6, this methodology has not only been been successfully used to produce additional stories for the personality trait self-esteem, but also to express user attitudes and stressors experienced. The more general methodology is the same as we used for personality (see Fig. 1), now using stories to express any characteristic.

There are several limitations and opportunities for future work. Firstly, the personality stories developed in this paper only portray a single trait. Although this enables investigations of the impact of such a trait, e.g on feedback to a learner, this does not facilitate investigations into interaction effects of multiple traits. To investigate this, stories which express two or more traits at the same time need to be developed.

Secondly, the stories developed in this paper only portrayed personality traits. We discussed above how the same method for constructing and validating stories has been used by us to portray other user and context characteristics such as stressors and user attitudes. We would like to extend this work by developing validated stories for portraying affective state, based on existing self-reporting affect scales. Similarly, we are interested in developing stories that reliably express other aspects such as learner performance and learner effort (a starting point towards the latter has been made in Okpo et al. (2017). When constructing such stories, care needs to be taken to avoid unintentionally evoking personality. For example, a learner who always performs well could be perceived as being highly conscientious, even when this was not the case. Another interesting area for validated story development may be to portray cultural differences (in line with Hofstede’s work on cultural dimensions Hofstede 1983).

In summary, whilst there has been substantial research effort on obtaining user-personality, there has been only very limited work on reliably expressing user personality. This paper has provided a methodology for doing so through validated personality stories, and has also shown that these stories can be used as an additional light-weight method for obtaining user personality.

## Footnotes

1 http://www.chambers.co.uk.

2 Personality is only one of many user characteristics that may impact user behaviour (Okpo et al. 2018). Other user characteristics include cognitive and physical ability (Loitsch et al. 2017), knowledge (Pelánek 2017), interests (Piao and Breslin 2018), and affect (Mizgajski and Morzy 2018; Grawemeyer et al. 2017). Additionally, situational factors, norms, and roles may moderate the impact of personality (Harland et al. 2007). Researchers normally investigate adaptation to personality together with other factors.

3 *vs* ‘Neuroticism (**N**)’. Referring to this trait in this way is more consistent with the nomenclature of the other four traits (with higher scores inferring more “positive” personalities), and removes the need to invert this score’s trait in analysis.

4 Based on the work by Perloff (2010), future work may include adapting to other personality traits such as self-monitoring, need for cognition, dogmatism and argumentativeness.

5 This is not an exhaustive list of traits, but a selection intended to convey the methodology, that we required for our other research.

6 Reproduced here to clarify how the stories were created; please refer to the original paper Schwarzer and Jerusalem (1995) when using the questionnaire.

7 This research was developed in the e-learning domain, where previous literature identified GSE, FFM and locus of control as salient adaptation characteristics. Therefore it would be desirable to have stories that isolated these traits, hence their inclusion.

8 As explained later, these stories needed alterations, and therefore crowd-sourcing was used to recruit the much larger number of participants required. The use of crowd-sourcing meant that we no longer investigated how the FFM stories were rated on the GSE and LOC scales, as including these scales would make the experiments too cumbersome and time consuming for participants on this platform.

9 Throughout this paper we use parametric measures to analyse Likert data. The conventional way to analyse personality tests is to total or average the score for the questions that relate to each factor; this indicates that the developers of these validated questionnaires intend the Likert scale items to be treated as numerical items. Indeed, the analyses of these questionnaires are generally provided by the scale developer using parametric methods. Whether to use a Mann–Whitney or *t* test on Likert data is debatable; Likert scales are commonly analysed using a *t* test, though there is good reason to treat them as non-parametric data. However, in practical application it has been found that there is little to no difference in the outcome, especially in the likelihood of Type 1 error (De Winter and Dodou 2010).

10 The ‘liked peer’ data was used as it was closer to the task in our experiment i.e. rating the personality of another person. A retrospective comparison of the self-reported minimarker scores of a subset of MTurk users revealed that the means for each trait are within 1SD of the means for the Illinois population, except for Emotional Stability, where the mean in the MTurk group was higher (6.29 vs 4.90). This is sufficiently similar to make the populations comparable.

11 The Minimarker scale was done first, to reduce the risk of straight-lining due to tiredness. There may have been a slight order effect, however as personality is relatively stable we do not expect an impact, particularly given the stories were not constructed from the Minimarker scale.
